# The Protective Effect of Intrasplenic Transplantation of Ad-IL-18BP/IL-4 Gene-Modified Fetal Hepatocytes on ConA-Induced Hepatitis in Mice

**DOI:** 10.1371/journal.pone.0058836

**Published:** 2013-03-13

**Authors:** Xueting Shao, Yun Qian, Chenhuai Xu, Bo Hong, Wanhong Xu, Ling Shen, Changzhong Jin, Zhigang Wu, Xiangmin Tong, Hangping Yao

**Affiliations:** 1 State Key Laboratory for Diagnosis and Treatment of Infectious Diseases, The First Affiliated Hospital, Zhejiang University School of Medicine, Hangzhou, Zhejiang, China; 2 Department of Pathology, The Second Affiliated Hospital, Zhejiang University School of Medicine, Hangzhou, Zhejiang, China; 3 Hangzhou High Throughput Drug Screening Center, ACEA Bio, Hangzhou, Zhejiang, China; French National Centre for Scientific Research, France

## Abstract

**Background:**

Concanavalin A (ConA)-induced hepatitis is an experimental murine model mirroring the pathology of human autoimmune hepatitis.

**Aim:**

To investigate the effects of intrasplenically transplanted fetal hepatocytes (BNL.CL2) transfected with recombinant adenovirus vector expressing the IL-18 binding protein (IL-18BP) and IL-4 fusion protein on ConA-induced hepatitis in mice.

**Methods:**

Ad-IL-18BP/IL-4 was used to infect BNL.CL2 cells. IL-4 and IL-18BP fusion protein expression were detected by ELISA and Western blotting. BNL.CL2 cells infected with Ad-IL-18BP/IL-4 were intrasplenically transplanted into mice. After 10 days, mice were injected with ConA (15 mg/kg)**, and** sacrificed 18 hours later. Liver injury was assessed by serum transaminase and liver histology. TNF-α, IL-18, IL-4, IL-10, IL-12p70 and monocyte-chemoattracting protein (MCP)-1 levels in serum and liver homogenates were detected by ELISA. Signaling molecules in liver homogenates were analyzed by Western blotting.

**Results:**

Ad-IL-18BP/IL-4 effectively expressed the IL-18BP/IL-4 fusion protein for more than 14 days in BNL.CL12 cells. Treatment of mice with Ad-IL-18BP/IL-4-BNL.CL2 before ConA injection significantly reduced the elevated plasma levels of transaminases compared with ConA control groups. TNF-α, IL-18, IL-12p70 and MCP-1 levels in serum and liver homogenates from mice transplanted with Ad-IL-18BP/IL-4-**BNL**.CL2 were lower and IL-4 and IL-10 levels were higher than control groups. Phosphorylation levels of NF-κB p65, AKT, p38 and JNK1/2 in liver homogenates were markedly suppressed by Ad-IL-18BP/IL-4.

**Conclusions:**

Ad-IL-18BP/IL-4 was effectively transfected into mouse BNL.CL2 cells. Intrasplenic transplantation of Ad-IL-18BP/IL-4-BNL.CL12 cells alleviated the severity of inflammation in ConA-induced experimental hepatitis and provides a useful basis for the targeted **gene therapy** of liver disease.

## Introduction

Concanavalin A (ConA) is a lectin (carbohydrate-binding protein) originally extracted from the jack-bean, *Canavalia ensiformis*. ConA is a hemagglutinin and a mitogen which is known for its ability to stimulate mouse T-cell subsets giving rise to four functionally distinct T-cell populations, including precursors to suppressor T-cells. It causes acute inflammation of the liver by the infiltration of activated lymphocytes, resulting in massive necrotic tissue injury of hepatocytes and intrasinusoidal hemostasis [1]. ConA-induced hepatitis in mice is commonly employed as an acute model for human autoimmune hepatitis because it mimics many aspects of this disease, including markedly increased serum levels of alanine transaminase (ALT) and inflammatory responses [2]. Compared to other liver injury models, ConA-induced hepatitis is characterized by necrotic injury, T-lymphocyte activation and proinflammatory cytokine overproduction [3]. Although the tissue injury caused by ConA is limited to the liver [4], the underlying mechanism that explains such organ specificity is still unclear.

IL-4 and IL-18 are considered essential for the development of ConA-induced hepatitis [5], [6]. However, previous studies have also demonstrated that ConA-induced liver injury can be suppressed by IL-4 [7]. IL-4 is a pleiotropic cytokine that plays a number of important roles, including the regulation of inflammation [8]. IL-4 acts as an autocrine growth factor promoting the differentiation of naive T cells to Th2 cells. It also inhibits the differentiation of naive T cells to Th1 cells as well as inhibiting cytokine production by Th1 cells [9]. IL-18 is a cytokine with powerful Th1-promoting activity in synergy with IL-12, and is a product of activated macrophages or Kupffer cells. IL-18 induces proliferation, up-regulates IL-2 receptor antagonist (IL-2Ra) expression, promotes IFN-γ, TNF-α and granulocyte-macrophage colony-stimulating factor (GM-CSF) production by Th1 clones and plays an important role in the induction of Thl response [10]. IL-18 binding protein (IL-18BP) is a member of a novel family of soluble proteins that also includes several poxvirus-encoded putative proteins. It is constitutively expressed in lymphoid tissues and can bind to IL-18, thus blocking its biological activity and limiting the contribution of IL-18 to Th1 responses [11]. As a consequence of their lack of stimulatory effects on immune cells, we have utilized the gene transfer of adenoviral vectors encoding IL-18BP and IL-4 in previous experiments, and shown this to be effective in animal models of immune inflammatory diseases, such as arthritis [12], [13]. Recombinant adenovirus vector is not integrated into the genome of the host cell, while adeno-associated vectors (AAV) are integrated, with the a potential risk of distortion [14]. These studies prompted us to investigate the effect of IL-18BP and IL-4 on ConA-mediated liver injury. Although the injections with recombinant IL-4 and IL-18BP proteins were efficient, recombinant proteins could be easily degraded and inactivated in vivo and multiple injections are often needed. In addition, the preparation and purification of recombinant protein is more costly. Thus, we used adenoviral vectors that encoded IL-18BP and IL-4 in our investigations.

## Materials and Methods

### Animals, Cell Lines and Antibodies

Male BALB/c mice aged 6–8 weeks were purchased from B&K Universal Group Limited (Shanghai, China) and housed in a temperature- and humidity-controlled environment. BNL.CL2, a normal embryonic mouse hepatic cell line, was obtained from the American Tissue Type Culture Collection (ATCC, Manassas, VA, USA). AD293 cells (adenoviral E1-transformed human embryonic kidney cells) were purchased from Stratagene (La Jolla, CA, USA). Enzyme-linked immunosorbent assay (ELISA) kits specific for mouse TNF-α, IL-18, IL-4, IL-10, IL-12p70 and MCP-1 were purchased from R&D Systems (Minneapolis, MN, USA). Mouse antibody specific for IL-18BP was obtained from R&D Systems and used as previously described [13]. HRP-conjugated goat anti-rabbit IgG antibodies were purchased from Santa Cruz Biotechnology (Santa Cruz, CA, USA). Rabbit antibodies specific for GFP, NF-κB p65, phospho-NF-κB p65, p38, phospho-p38, JNK1/2, phospho-JNK1/2, Akt and phospho-Akt and HRP-conjugated rabbit anti-GAPDH monoclonal antibody were purchased from Cell Signaling Technology (Danvers, MA, USA). RPMI-1640 medium and fetal calf serum (FCS) were purchased from Gibco (Invitrogen, Grand Island, NY, USA).

### Recombinant Adenovirus Preparation

Replication-defective recombinant adenoviruses Ad-IL-18BP/IL-4 and Ad-IL-18BP/IL-4-GFP encoding murine IL-18BP/IL-4 fusion protein, and Ad-LacZ and Ad-LacZ-GFP encoding β-galactosidase were constructed as previously described [12], [13]. These adenoviruses were propagated in AD293 cells and the titers of the adenoviruses were determined by using a standard plaque-forming unit assay [12].

### Gene Modification of Hepatocyte Cell Line *in* *vitro*

Gene modification of hepatocyte cell lines *in vitro* was performed as previously described [12]. Briefly, BNL.CL2 cells were washed twice in RPMI-1640 and resuspended at 1×10^6^ cells/mL. These cells were then infected with recombinant adenovirus at multiplicities of infection (MOI) from 5 to 100. After incubation at 37°C for 4 hours, the culture medium was replaced to remove free viral particles, the cells were washed twice in PBS and then adjusted to 2×10^7^ cells/mL for use in intrasplenic transplantation. For examination of IL-18BP and IL-4 expression by Ad-IL-18BP/IL-4-BNL.CL2 cells, infected cells were washed, adjusted to 1×10^6^cells/mL and maintained in fresh RPMI-1640 complete medium (supplemented with 10% FCS, 100 U/mL penicillin, and 50 µg/mL streptomycin). Culture supernatants were collected at different time points and the IL-4 concentration measured by ELISA (R&D Systems, USA). Expression of green fluorescent protein (GFP) by BNL.CL2 cells transfected with Ad-IL-18BP/IL-4-GFP and Ad-LacZ-GFP was demonstrated using an Olympus 1×2-UCB microscope with a 100× objective to take fluorescence photographs.

### MTS Assay for BNL.CL2 Cell Activation

BNL.CL2 cells transfected with recombinant adenovirus for 30 minutes were seeded in triplicate into a 96-well plate at 2×10^4^/well. After 48 hours, 96 hours or 6 days, 20 µl CellTiter 96 AQueous One Solution Cell Proliferation Assay kit (MTS; Promega, Madison, WI, USA) was added to each well. Microplates were incubated at 37°C for an additional 4 hours. Absorbance at 490 nm was measured using a Model 680 microplate reader (Bio-Rad, Hercules, CA, USA).

### Western Blotting

Samples containing 1×10^6^ cells or 100 mg tissue were lysed in 200 µL cell lysis buffer (Cell Signaling Technology) for 30 minutes at 4°C, and then clarified by centrifugation at 8,000 *g* for 10 minutes. Protein concentration was determined using a BCA protein assay kit (Pierce, Rockford, IL, USA), with bovine serum albumin as the standard.

Western blotting was performed as described previously [13]. Briefly, cell lysates were denatured for 10 minutes at 95°C in SDS-PAGE sample buffer, electrophoresed on 10% SDS-PAGE gels, and transferred to polyvinylidene difluoride (PVDF) membranes. Membranes were blocked with 5% nonfat milk in Tris-buffered saline and Tween 20 (TBST) and then incubated with specific antibodies for 2 hours at room temperature. After thorough washing, blots were incubated with HRP-conjugated secondary antibody. Protein band intensity was analyzed using enhanced ECL reagents (Amersham, Piscataway, NJ, USA) and a VersaDoc MP5000 imaging system (Bio-Rad).

### Animal Treatment

This study was carried out in strict accordance with the recommendations in the Guide for the Care and Use of Laboratory Animals of the National Institutes of Health. The protocol was approved by the Committee on the Ethics of Animal Experiments of Zhejiang University (Permit Number: 2012-172). All surgeries were performed under sodium pentobarbital anesthesia, and all efforts were made to minimize suffering.

**Figure 1:**
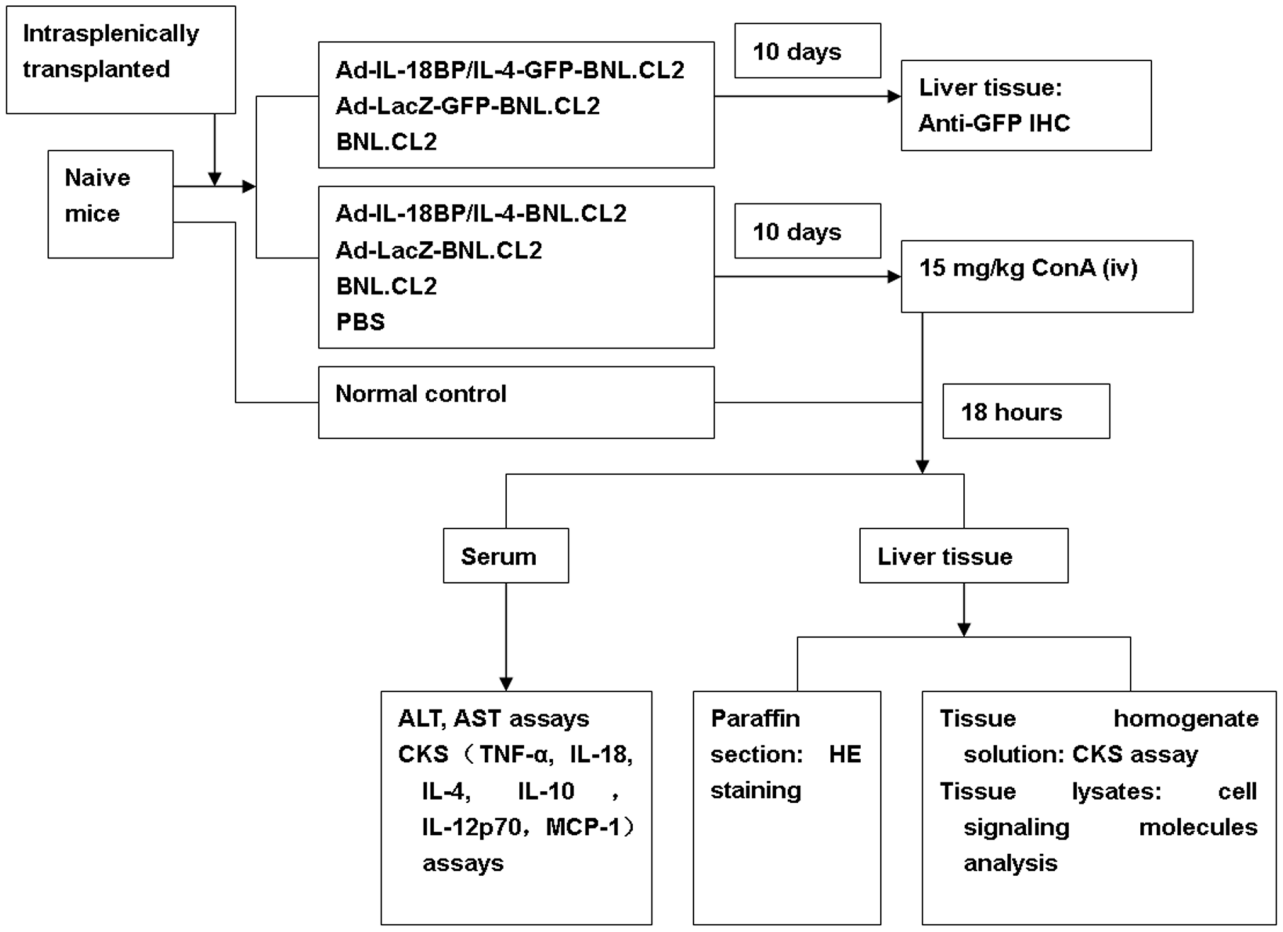
Experimental protocol. Ad-IL-18BP/IL-4, adenoviral vector encoding IL-18BP and IL-4; Ad-LacZ, adenoviral vector expressing β-galactosidase; ConA, concanavalin A; iv, intravenously; ALT; ALS; CKS; GFP; HE; IHC.

Six- to 8-week-old male mice weighing approximately 20 g were divided into seven groups (n = 6 mice in each group; Fig. 1) and intrasplenically transplanted (2×10^6^ cells/0.2 mL/mouse) with the IL-18BP/IL-4 gene-modified hepatocyte cell line or one of the other control cell lines (Ad-IL-18BP/IL-4-GFP-BNL.CL2, Ad-LacZ-GFP-BNL.CL2, Ad-LacZ-BNL.CL2 or BNL.CL2). All experiments were conducted as previously described [15]–[17]. For intrasplenic transplantation of BNL.CL2 cells, mice were anesthetized, a small surgical incision was made in the flank and the spleen was exposed. Freshly harvested BNL.CL2 cells (2×10^6^ BNL.CL2 cells with or without gene modification) suspended in PBS were injected into the inferior pole of the spleen using a 25-gauge needle connected to a 1-mL syringe, avoiding constriction of the hilar blood vessels. Homeostasis was secured with a ligature around the spleen proximal to the injection site.

Ten days later, the mice in each group (except the AdIL18BP/IL-4-GFP-BNL.CL2 and Ad-LacZ-GFP-BNL.CL2 groups) were injected via the tail vein with 1 mg/mL ConA (Sigma, St. Louis, MO, USA) in PBS administered at 15 mg/kg body weight. An untreated group served as a normal control. The mice were sacrificed 18 hours after ConA injection and serum samples were collected and frozen at −80°C until further analysis. Mouse livers were harvested for histopathological examination, extraction of liver tissue homogenate solution [18] or liver tissue lysates (Fig. 1).

### Preparation of Liver Homogenates

A 100-mg portion of the left lobe of the liver was homogenized in 0.5 mL PBS. After centrifugation at 12,000 rpm for 5 minutes, the supernatant was collected and stored at −20°C for measurement of cytokines.

### Measurement of Alanine Transaminase (ALT), Glutamic-oxaloacetic Transaminase (AST) and Cytokines

Serum levels of ALT and AST, and levels of TNF-α, IL-18, IL-4, IL-10, IL-12p70 and MCP-1 in serum and liver homogenates were detected by commercially available ELISA kits following the manufacturer’s instructions. Results were presented as U/mL or pg/mL.

### Immunohistochemical (IHC) Staining

Paraffin-embedded liver sections were deparaffinized and rehydrated using xylene and ethanol, respectively. IHC staining was performed on mouse tumor tissues as previously described [19]. Briefly, tissues were labeled using primary antibodies specific for green fluorescent protein (GFP), and antibody binding was detected using EnVision System reagents (DAKO, Tokyo, Japan).

### Statistical Analysis

The statistical significance of comparisons between groups was determined with a Q-test. P-values of less than 0.05 were considered statistically significant.

## Results

### The Expression of IL-18BP/IL-4 in Gene-modified BNL.CL2 Cells

**Figure 2:**
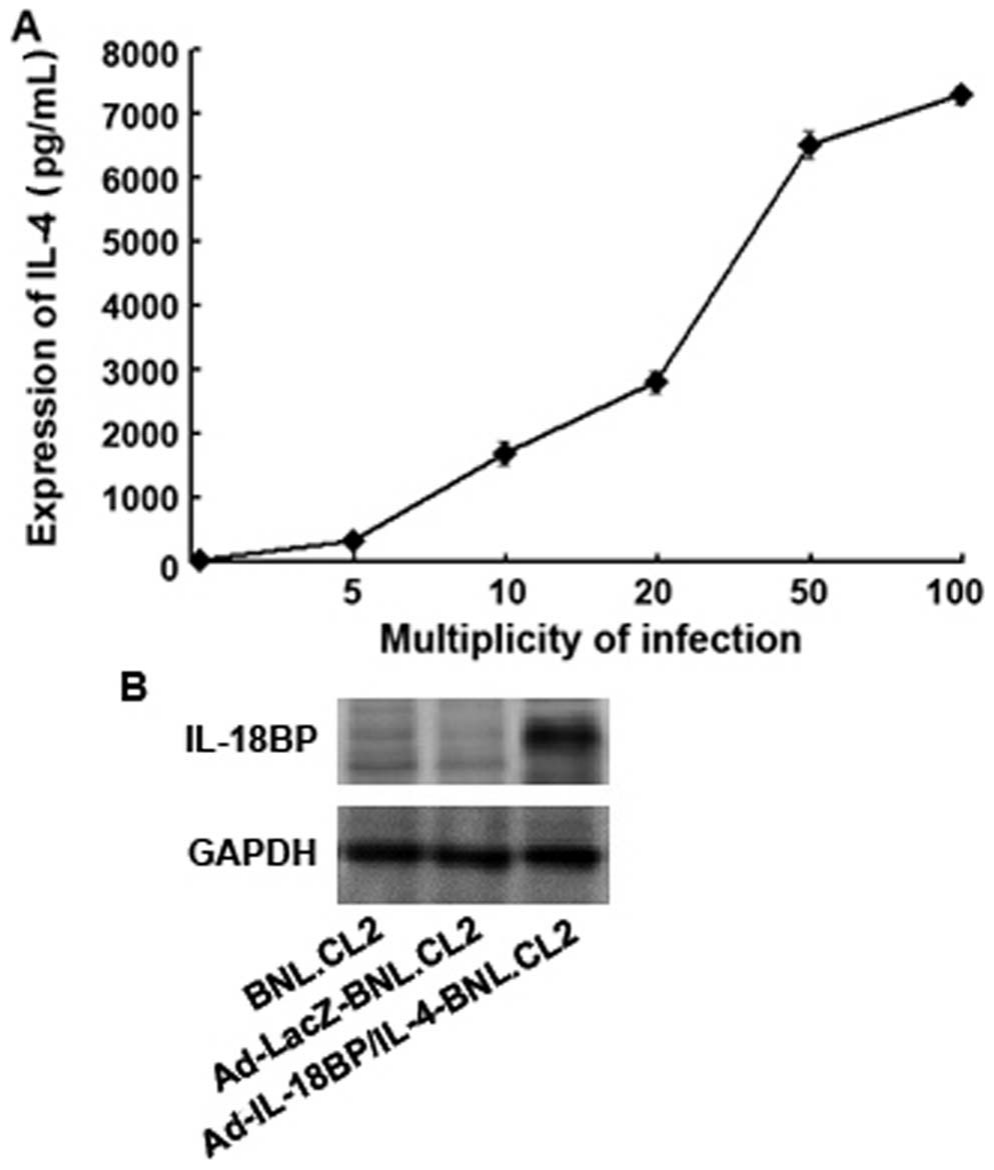
Expression of Ad-IL-18BP/IL-4 in BNL.CL2 cells. (**A**) The expression of IL-4 in BNL.CL2 cells transfected with Ad-IL-18BP/IL-4 for 48 hours at multiplicities of infection (MOI) from 5 to 100. IL-4 concentration in culture supernatants was analyzed by ELISA. (**B**) The expression of IL-18BP in BNL.CL2 cells transfected with recombinant adenovirus for 48 hours at an MOI of 50, determined by Western blotting. Data are from three independent experiments (A; mean ± sd) or are representative of three independent experiments with similar results (B).

In order to examine the *in vitro* expression of IL-18BP and IL-4 by IL-18BP/IL-4 gene-modified BNL.CL2 cells, culture supernatants were harvested 48 hours after transfection with recombinant adenoviruses at MOIs from 0 to 100, and IL-4 concentrations were measured by ELISA. We showed that the expression of IL-4 secreted by Ad-IL-18BP/IL-4-BNL.CL2 cells increased with increasing MOI (Fig. 2A). This expression peaked at an MOI of 50, reaching a concentration of 6520 pg/mL, while there was no significant difference in expression between MOI of 50 and 100. IL-18BP was also detected in Ad-IL-18BP/IL-4-BNL.CL2, by Western blotting, while Ad-LacZ-BNL.CL2 and normal BNL.CL2 cells did not express IL-18BP (Fig. 2B). An MOI of 50 was chosen for all subsequent experiments.

**Figure 3:**
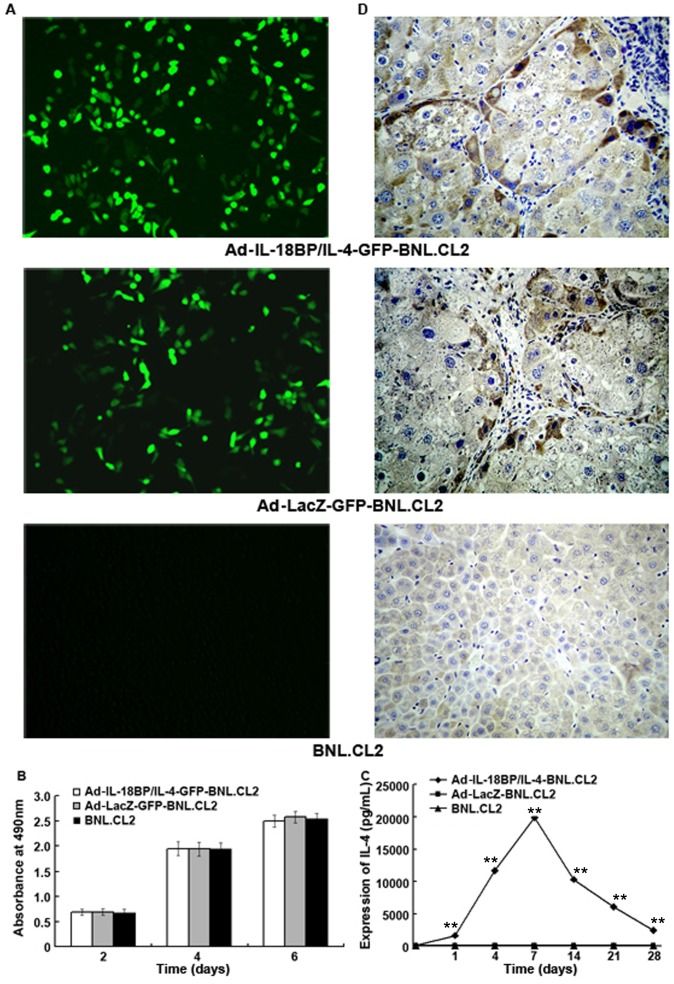
Expression of Ad-IL-18BP/IL-4-GFP in BNL.CL2 cells. (**A**) The expression of green fluorescent protein (GFP) in BNL.CL2 cells transfected with Ad-IL-18BP/IL-4-GFP for 48 hours (100×). (**B**) BNL.CL2 cell proliferation was determined by MTS 2, 4 and 6 days after transfection with Ad-IL-18BP/IL-4 for 48 hours. Data points indicate absorbance at 490 nm. (**C**) The expression of IL-4 by BNL.CL2 cells transfected with Ad-IL-18BP/IL-4 at an MOI of 50. Culture supernatants were analyzed by ELISA at time points from 1 to 28 days. (**D**) Mice were killed 10 days after intrasplenic transplantation of BNL.CL2 transfected with Ad-IL-18BP/IL-4-GFP or Ad-LacZ-GFP, and liver samples were prepared for detection of GFP expression using immunohistochemistry (200×). **P<0.01 vs. Ad-LacZ-BNL.CL2 and BNL.CL2. Data are representative of three independent experiments with similar results (A and D) or are from three independent experiments (B and C; mean ± sd).

Ad-IL-18BP/IL-4-GFP and Ad-LacZ-GFP were constructed as controls for Ad-IL-18BP/IL-4 and Ad-LacZ to observe the transfection efficiency of recombinant adenovirus in BNL.CL2 cells. Figure 3A shows that the recombinant adenoviruses had high transfection efficiency in BNL.CL2 cells 48 hours after transfection. Ad-IL-18BP/IL-4 and Ad-LacZ had no effect on cell activation, since there was no significant difference in absorbance at 490 nm in an MTS assay between Ad-IL-18BP/IL-4-BNL.CL2, Ad-LacZ-BNL.CL2 and normal BNL.CL2 cells (Fig. 3B). Culture supernatants were harvested at different time points for measurement of IL-4 concentration by ELISA. Figure 3C shows that 24 hours after transfection, Ad-IL-18BP/IL-4-BNL.CL2 cells had begun to secrete detectable levels of IL-4 and that this expression peaked at 7 days, reaching a concentration of 19876 pg/mL. Ad-IL-18BP/IL-4-BNL.CL2 cells continued to express significant levels of IL-4 (2415 pg/mL) until 28 days after transfection, while adenovirus-mediated *LacZ* gene-modified BNL.CL2 (Ad-LacZ-BNL.CL2) and normal BNL.CL2 cells expressed very low levels of IL-4 over this time. The results suggest that BNL.CL2 could be effectively transfected by Ad-IL-18BP/IL-4 and secrete IL-4 *in vitro* for at least 28 days.

To examine the *in vivo* expression of recombinant adenovirus in normal, healthy mice after intrasplenic transplantation, mice were killed 10 days after transplantation of BNL.CL2 cells transfected with Ad-IL-18BP/IL-4-GFP or Ad-LacZ-GFP, and liver samples were prepared for detection of GFP expression using IHC. As shown in Fig. 3D, GFP expression was detected in mouse liver tissues following intrasplenic transplantation with Ad-IL-18BP/IL-4-GFP-BNL.CL2 or Ad-LacZ-GFP-BNL.CL.2, but not with normal BNL.CL2 cells. These data indicate that Ad-IL-18BP/IL-4 gene-modified BNL.CL2 cells, after intrasplenic transplantation, were able to express IL-18BP/IL-4 *in vivo* in the liver.

### The Regulatory Effects of IL-18BP/IL-4 Gene-modified BNL.CL2 Cells on Cytokines in Mice with ConA-induced Hepatitis

IL-18 and IL-4 are known to be important cytokines, and both have an immunoregulatory function in the differentiation of T subsets [20], [21]. We therefore examined the influence of intrasplenic transplantation of Ad-IL-18BP/IL-4-BNL.CL2 cells on cytokine expression in peripheral blood and liver in mice with ConA-induced hepatitis. Mice from different groups were killed 18 hours after injection with ConA (Fig. 1), and samples were prepared as described in the Materials and Methods.

**Figure 4:**
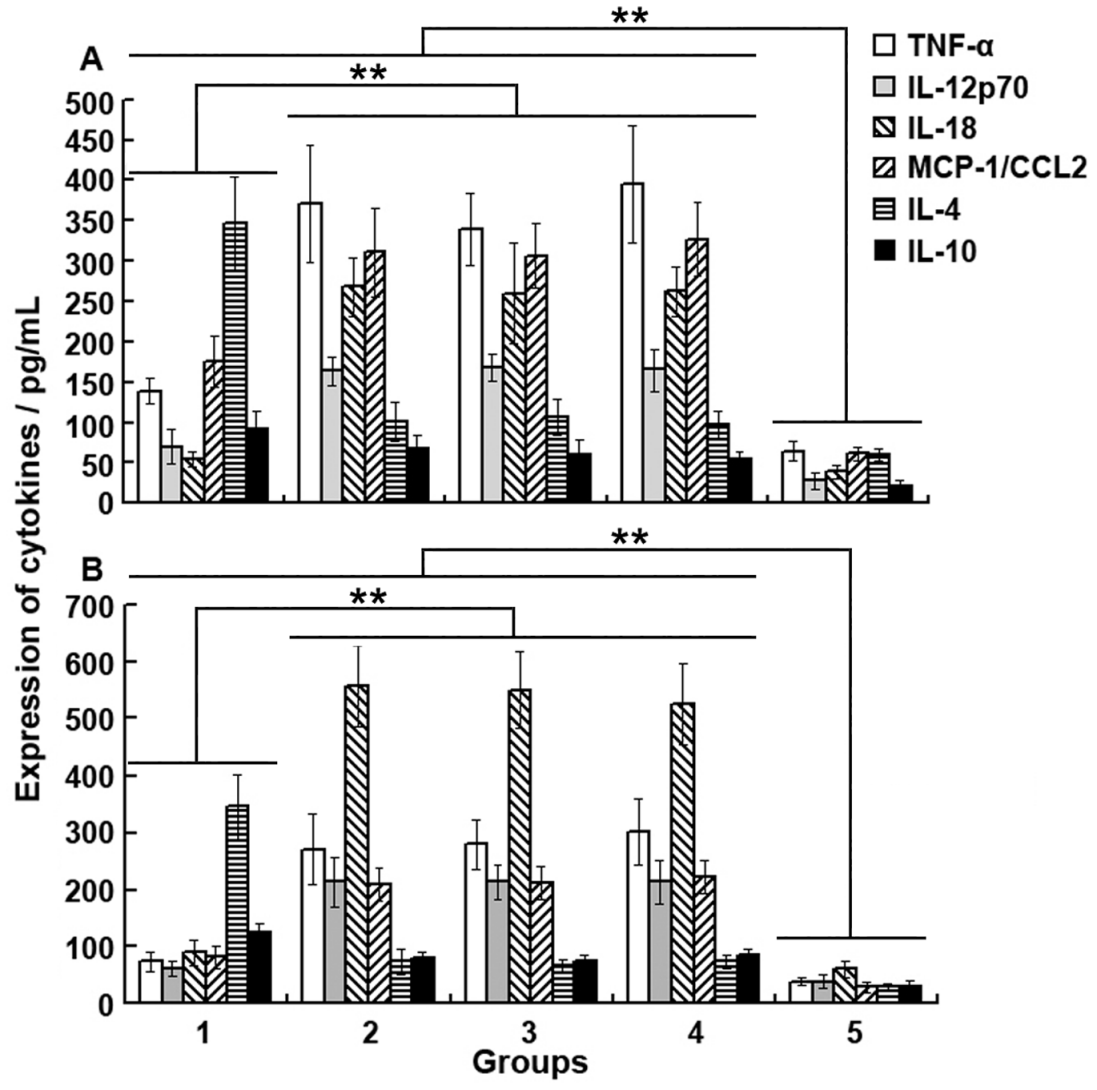
Expression of cytokines in mice with ConA-induced hepatitis pretreated with IL-18BP/IL-4 gene-modified hepatocytes. Ten days after intrasplenic transplantation of Ad-IL-18BP/IL-4-BNL.CL2 or control cells, mice from different groups were injected with ConA, killed 18 hours later and samples were prepared as described in the Materials and Methods. ELISA was used to measure the expression of cytokines in (**A**) liver homogenates and (**B**) serum from mice in the following groups: Group 1, Ad-IL-18BP/IL-4-BNL.CL2+ ConA; Group 2, Ad-LacZ-BNL.CL2+ ConA; Group 3, BNL.CL2+ ConA; Group 4, ConA; Group 5, normal control. **P<0.01. Data are from one experiment representative of three independent experiments with similar results (mean ± sd of six samples).

Figure 4A shows the influence of intrasplenic transplantation of Ad-IL-18BP/IL-4-BNL.CL2 cells on the expression of cytokines in the liver. Our data shows that concentrations of IL-4 and IL-10 in liver homogenates of the mice intrasplenically transplanted with Ad-IL-18BP/IL-4-BNL.CL2 cells and then injected with ConA (Ad-IL-18BP/IL-4-BNL.CL2+ ConA group) were increased significantly when compared with those of the Ad-LacZ-BNL.CL2+ ConA, BNL.CL2+ ConA and ConA control groups. In contrast, concentrations of TNF-α, IL-18, IL-12p70 and MCP-1 were significantly decreased (*P*<0.01) in the Ad-IL-18BP/IL-4-BNL.CL2+ ConA group when compared with those in the three ConA control groups, while still a little higher than in the normal group. Figure 4B shows similar results for the serum concentrations of cytokines in these mice. Mice with intrasplenically transplanted Ad-IL-18BP/IL-4-BNL.CL2 cells showed significantly increased serum concentrations of IL-4 and IL-10, and decreased concentrations of TNF-α, IL-18, IL-12p70 and MCP-1 (*P*<0.01), in a similar pattern to that of the liver homogenates.

### The Protective Effect of Ad-IL-18BP/IL-4 Against ConA-induced Liver Injury in Mice

**Figure 5:**
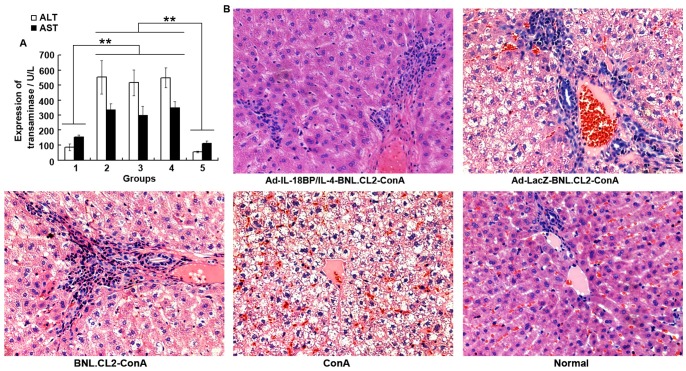
The protective effect of Ad-IL-18BP/IL-4 against liver injury in mice. (**A**) Mouse peripheral blood was harvested 18 hours after ConA injection and ALT and AST activity was analyzed by ELISA. (**B**) Mouse livers were harvested 18 hours after ConA injection for histopathological examination staining with hematoxylin-eosin staining (HE). Group 1: Ad-IL-18BP/IL-4-BNL.CL2+ConA; Group 2: Ad-LacZ-BNL.CL2+ConA; Group 3: BNL.CL2+ConA; Group 4: ConA; Group 5: normal control (200×). **P<0.01. Data are from one experiment representative of three independent experiments with similar results (mean ± sd of six samples).

Transaminase is an important indicator of liver function. We therefore examined the effects of intrasplenic transplantation with Ad-IL-18BP/IL-4-BNL.CL2 cells on the production of ALT and AST. As shown in Fig. 5A, the concentrations of ALT and AST in the peripheral blood of mice intrasplenically transplanted with Ad-IL-18BP/IL-4-BNL.CL2 cells were significantly lower than in the three control groups (*P*<0.01), and were similar to those in the normal group. Liver sections stained with hematoxylin and eosin (HE) showed that massive necrosis of the liver was present in mice treated with Ad-LacZ-BNL.CL2+ ConA, BNL.CL2+ ConA or ConA alone (Fig. 5B), but that Ad-IL-18BP/IL-4-BNL.CL2 pretreatment reduced the ratio of necrotic to total tissue area by 69.1% compared with Ad-LacZ-BNL.CL2+ ConA treated mice (*P*<0.05). This result is consistent with the decreased levels of ALT and AST in this group, and suggests a protective effect of Ad-IL-18BP/IL-4 against liver injury.

### The Signaling Molecules Involved in the Protective Effect of Ad-IL-18BP/IL-4

**Figure 6:**
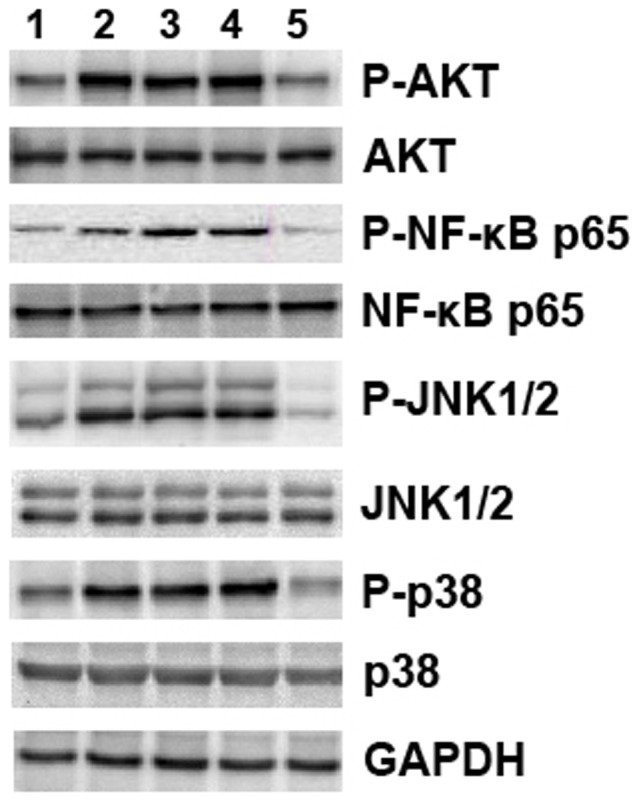
Signaling molecules involved in the protective effect of Ad-IL-18BP/IL-4. Mouse livers were harvested 18 hours after ConA injection. Liver tissue lysates were used for Western blotting with primary antibodies to NF-κB p65, AKT, p38, JNK1/2 and their phosphorylated counterparts. Lane 1: Ad-IL-18BP/IL-4-BNL.CL2+ConA; Lane 2: Ad-LacZ-BNL.CL2+ConA; Lane 3: BNL.CL2+ConA; Lane 4: ConA; Lane 5: normal control. Data are from one experiment that was representative of three independent experiments that had similar results.

Figure 6 shows that the phosphorylation levels of NF-κB p65, AKT, p38 and JNK1/2 in liver homogenates were markedly suppressed by Ad-IL-18BP/IL-4 compared with the Ad-LacZ-BNL.CL2+ConA, BNL.CL2+ConA and ConA control groups.

## Discussion

Targeted gene therapy is a current research focus. Gene transfer into donor cells *in vitro* followed by transplantation into the same subject or allografting into immunologically unrelated subjects constitutes *ex vivo* targeted gene therapy. Liver-targeted *ex vivo* gene therapy using hepatocytes as recipient cells has been applied to a number of diseases of the liver. Cao et al. [15] found that genetically modified mouse hepatocytes could be targeted to the liver via intrasplenic transplantation and could play an immunoregulatory role. Hepatocytes which are intrasplenically transplanted can rapidly migrate to and randomly distribute throughout the liver, and are capable of long-term survival with no significant side effects [22]. It has been demonstrated that intrasplenic transplantation of hepatocytes efficiently expressing a therapeutic gene *in vivo* is an effective strategy for the treatment of acute or chronic liver disease [23]. It has also been reported that gene-modified hepatocytes transplanted intrasplenically into mice migrated to the liver within 24 hours after transplantation and expressed the exogenous gene for 11 weeks [17], and that intrasplenic transplantation of gene-modified hepatocytes effectively activated liver immune function and exerted potent therapeutic effects on liver disease in mice [15]. BNL.CL2 is a normal mouse embryonic hepatic cell line. BNL.CL2 cells were appropriate for our research for several reasons, such as the consistent cellular characteristics, their rapid rate of growth and the fact that these cells were easy to culture and obtain [16]–[18]. However, the vigor of the primary mouse hepatocytes that were prepared freshly was markedly lower than cultured cell lines, and it was difficult to confirm their purity with noticeable differences between different batches. Moreover, the proliferation activity of primary mature mouse hepatocytes was low in vitro and the expression of recombinant adenovirus gene in the cells was low, transient and easily deactivated. The use of mouse fetal hepatocytes as carriers bypassed the shortcomings of primary hepatocytes.

In addition, our previous studies showed that recombinant adenovirus vector Ad-IL-18BP/IL-4 was able to inhibit the inflammatory response [13]. Therefore, the primary objective of the present study was to investigate the regulatory effects of intrasplenic transplantation of IL-18BP/IL-4 gene-modified BNL.CL2 cells on the hepatic and systemic immune state in mice with ConA-induced hepatitis and to observe the anti-inflammatory effects of this therapy on hepatitis in the murine model. Very little adenovirus will reach the liver via intravenous injection, and the adenovirus vector will be rapidly degraded. Compared to such methods, the use of gene-modified fetal hepatocytes via intrasplenic transplantation was a better targeting tool.

ALT and AST activity in serum and liver histopathology are widely used as conventional indicators for the evaluation of liver injury [24]. In our study, Ad-IL-18BP/IL-4 delivery significantly decreased hepatic injury, as judged by reduced serum ALT and AST activity, histopathological changes and a reduction in the relative area of necrotic liver tissue.

It has been demonstrated that proinflammatory mediators are involved in ConA-induced liver damage. ConA stimulation has been reported to significantly increase TNF-α [25], IL-18 [5], IL-12p70 [26] and MCP-1 [27] in serum, and depletion of these mediators decreases hepatic damage. TNF-α is a proinflammatory cytokine that plays a crucial role in the response to tissue injury, infection, and inflammation [28]. TNF-α causes acute inflammatory hepatocellular apoptosis followed by organ failure and is the central mediator in several experimental models of hepatotoxicity [29]. The mechanisms of action of endogenous and exogenous IL-4 on inflammation *in vivo* are not clear. Previous studies suggest that a possible explanation for the tissue-protective effect of IL-4 might be its immunoregulatory ability to play a critical role in the Th2 reaction and to modulate the IL-1- and TNF-α-mediated inflammatory responses [30]. IL-1 has also been proven to be a critical cytokine involved in ConA-induced hepatitis [31]. An imbalance in this system exists because the relative levels of production of IL1 receptor antagonist (IL-1Ra) are not adequate to effectively block the proinflammatory effects of IL-1 [32]. Some studies have revealed that IL-4 treatment in animal models can enhance levels of IL-1Ra or suppress levels of IL-1β [33]. The current study has shown in mouse ConA-induced hepatitis that IL-4 gene delivery resulted in anti-inflammatory effects *in vivo,* reducing liver injury through the inhibition of proinflammatory cytokines. This study implied that IL-4 gene therapy might be a useful approach to the reduction of inflammation in hepatitis.

The production of IL-10 is one of the anti-inflammatory mechanisms used to control a dysregulated immune response in reaction to the activity of proinflammatory cytokines. IL-10 can suppress TNF-α and IL-1 production by activated macrophages and can enhance the production of TNF inhibitors, acting as a potent anti-inflammatory cytokine [34]. IL-4 helps B cells to produce more IL-10 and therefore skews the immune response toward Th2 cells [35]. IL-12 is a dominant cytokine involved in the development of IFN-gamma-producing T cells, promoting the Th1 type response. IL-4 can strongly inhibit IL-12, resulting in a negative feedback effect on the proliferation of Th1 cells [36]. MCP-1 is a chemokine with potent monocyte-activating and attracting properties [37]. The important role of MCP-1 during inflammation has been demonstrated in recent studies showing markedly increased tissue levels during inflammatory bowel disease [38]. It has been reported that IL-4 can downregulate MCP-1 production in activated intestinal epithelial cells [39]. In our study, IL-4 shifted the balance of T cell responses from Th1 to Th2, and decreased MCP-1 to inhibit the attraction and activation of monocytes. IL-4 therefore has the potential to reduce inflammation in ConA-induced hepatitis.

We also found that serum levels of IL-18 were significantly downregulated in response to local Ad-IL-18BP/IL-4 treatment in mice with hepatitis. IL-18, a pleiotropic cytokine produced by activated macrophages, plays an important role in the Th1 response [10]. IL-18 synergizes with IL-12 to induce the production of IFN-γ and potentiates Th1 and natural killer cell cytotoxicity by augmenting Fas ligand- and perforin-mediated cytotoxic activity. IL-18 also plays a critical role in TNF-α- and Fas ligand-mediated liver injury [10]. IL-18BP is a naturally occurring protein that binds and neutralizes IL-18. IL-18BP binds IL-18 with specificity and high affinity and blocks its biological activities [11]. Such a naturally occurring molecule represents an interesting inhibitor for testing in experimental models of disease. IL-18 is an early signal leading to Th1 cytokine responses that are essential for the cytotoxic T cell response. IL-18BP could therefore potentially modulate one of the earliest phases of the Th1 immune response [11]. Moreover, local overexpression of IL-18BP by adenoviral delivery has been shown to ameliorate tissue destruction in a model of arthritis [40]. The therapeutic use of IL-18BP might therefore modulate the cytokine balance, ameliorating established hepatitis. Our data show for the first time the marked effect of modifying Th1/Th2 imbalance in ConA-induced hepatitis mice after local Ad-IL-18BP/IL-4 gene therapy.

We next focused on the mechanism underlying the decrease in Th1-type cytokines and the increase in Th2-type cytokines in response to Ad-IL-18BP/IL-4. Intracellular signal pathways via Akt, MAPK (p38 MAPK and JNK1/2) and NF-κB play crucial roles in the regulation of cytokine production. It has been reported that the PI3K-Akt pathway plays a pivotal role in regulating production of inflammatory mediators in both human and mouse monocytes and macrophages [41], while its activation can promote the activation of NF-κB p65 [42]. Additionally, the ability of PI3K to influence NF-κB p65 may be related to its ability to activate p38 [43]. The p38 MAPK, NF-κB and JNK1/2 pathways are responsible for the production of inflammatory cytokines, including TNF-α, IL-18, IL-4, IL-10 and IL-12 [44]–[47]. We found that Ad-IL-18BP/IL-4 treatment resulted in marked attenuation of Akt, p38 MAPK, NF-κB p65 and JNK1-2 activity in ConA-induced hepatitis.

### Conclusions

Ad-IL-18BP/IL-4-BNL.CL2 pretreatment significantly alleviated ConA-induced liver injury in mice. The protective effects of Ad-IL-18BP/IL-4 on hepatotoxic responses to ConA were closely related to the inhibition of Th1 cell activation and proinflammatory cytokine release through the NF-κB p65, AKT, p38 and JNK1/2 pathways. Our results strongly suggest that IL-18BP/IL-4 gene transfer can prevent ConA-mediated hepatitis and liver injury.
